# C-Reactive Protein Levels and Clinical Prognosis in LAA-Type Stroke Patients: A Prospective Cohort Study

**DOI:** 10.1155/2021/6671043

**Published:** 2021-06-08

**Authors:** Qingjia Zeng, Yaying Zeng, Mark Slevin, Baoqiang Guo, Zhipeng Shen, Binbin Deng, Wenbo Zhang

**Affiliations:** ^1^University College London, Institute of Health Informatics, UK; ^2^Department of Neurology, The First Affiliated Hospital of Wenzhou Medical University, Wenzhou, China; ^3^Department of Life Science Faculty of Science and Engineering, Manchester Metropolitan University, Manchester M1 5GD, UK; ^4^Department of Neurosurgery, The Children's Hospital of Zhejiang University School of Medicine, National Clinical Research Center for Child Health, Hangzhou, China

## Abstract

**Methods:**

We prospectively included 200 patients with LAA-type AIS and tested their CRP levels on admission. We followed these patients consecutively. The primary outcome was an adverse event, defined as a modified Rankin Scale score of 2-6 at months 3, 6, and 12 after discharge. A logistic regression model was used to analyze the relationship between CRP and the functional outcome of LAA stroke.

**Results:**

We divided 200 patients into 3 groups evenly based on CRP level. After adjustment for gender, age, smoking history, drinking history, history of hyperlipidemia, history of diabetes, lipid levels, and blood glucose levels, logistic regression showed that the incidence of LAA-type AIS poor outcome was positively associated with CRP level at admission, whether it was 3 months, 6 months, or 12 months after discharge, respectively (OR: 2.574, 95% CI: 1.213-5.463; OR: 2.806, 95% CI: 1.298-6.065; OR: 2.492, 95% CI: 1.167-5.321. In the highest tertile vs. the lowest tertile as a reference), and both were statistically different.

**Conclusions:**

High CRP level predicts poor functional outcome in LAA-type AIS patients, which provides a strong basis for clinicians to make treatment decisions for these patients.

## 1. Introduction

Stroke is one of the most common causes of disability and death and is associated with a remarkable economic and social burden [1, 2]. Acute ischemic stroke (AIS) is the most common type of stroke, accounting for 87% of all strokes [3]. There is growing evidence that ischemic stroke is related to the systemic inflammatory response, and diverse inflammatory cytokines are being investigated as potential predictors of cardiovascular and functional prognosis after AIS [4, 5].

It is increasingly recognized that inflammation plays a central role in atherosclerosis and cardiovascular disease [6]. Inflammation plays a crucial role in the pathogenesis of ischemic stroke, with mechanisms of action including atherosclerosis, plaque instability, and plaque rupture triggering [7, 8]. In the case of an acute stroke, inadequate blood flow to the brain leads to an interruption in the supply of oxygen and glucose to the neurons, resulting in massive cell death within the infarcted core [9]. Damage signals are released from dying cells in the ischemic core and peri-infarct region [10]; these signals activate leukocytes and microglia, leading to a substantial release of proinflammatory cytokines, upregulate the expression of leukocyte adhesion molecules, and stimulate the formation of chemokines. Combined with increased blood-brain barrier (BBB) permeability, this allows leukocytes to enter the ventricles in large numbers and remove the large amount of debris caused by cell death [11, 12].

Among the inflammatory markers of peripheral blood, CRP is the most widely used and well established [12]. Previous studies have shown that in the general population, elevated serum or plasma CRP may lead to future vascular events [13–16]. A study reports high levels of CRP associated with clinical prognosis in the time window between 12 and 72 hours after ischemic stroke [17, 18]. CRP is of clinical importance as an early prognostic factor after stroke because it is an easily measured and clinically common indicator of inflammation. However, the vast majority of current studies address the relationship between AIS and CRP and are mostly retrospective. AIS has different subtypes, and large artery atherosclerosis (LAA) is a key subtype of the Trial of Org 10172 in Acute Stroke Treatment (TOAST) classification system. Elevated CRP often indicates a worse prognosis in AIS patients, but it is not clear whether it can predict the patient's functional outcome in LAA patients. We explored this topic.

## 2. Materials and Methods

### 2.1. Study Design

We enrolled patients with AIS between January 2016 and January 2017. Stroke is an acute cerebrovascular disease with acute or focal brain dysfunction caused by various vascular etiologies lasting longer than 24 hours. We use brain imaging data (computed tomography and/or magnetic resonance imaging) to diagnose AIS. According to TOAST criteria, AIS can be divided into four subtypes: LAA, small vessel occlusion, cardioembolic, and others [19]. This study included patients with LAA according to TOAST criteria.

### 2.2. Exclusion Criteria

The exclusion criteria are as follows: (1) excluding other vascular infarction, cerebral venous thrombosis; (2) on anti-inflammatory therapy prior to hospitalization; (3) any factor that would affect indicators of inflammation, including serious infection or use of antibiotics prior to admission, hematologic disorders, immune system disorders, glucocorticoid use, or severe liver or kidney disease, and recent trauma or major surgery; (4) patients lost to follow-up. All patients were admitted within 7 days of stroke onset, and their baseline data were collected using standardized data records. Therefore, 200 remained for analysis. We evenly divided 200 patients into 3 groups according to CRP from low to high. There were 66 patients, 67 patients, and 67 patients in each group. We defined them as group A, group B, and group C. In this way, we have 3 subgroups with different CRP levels and roughly equal numbers of patients.

### 2.3. Clinical and Laboratory Assessments

All blood indicators were assessed on the morning of the second day of admission after overnight fasting. CT examinations were completed within 24 hours of admission; confirmation of AIS relies on repeat CT and MRI exams prior to hospital discharge.

### 2.4. Follow-Up

The National Institutes of Health Stroke Scale (NIHSS) score was used for assessment at admission and on the first day after admission and at discharge. Patients who survived the acute phase and were discharged or hospitalized for rehabilitation were assessed for functional outcome every 3 months by follow-up using a modified Rankin Scale (mRS) score. Good outcomes were defined as mRS scores 0-1, while poor outcomes were defined as mRS scores 2-6. Two investigators evaluated all clinical data alone. In case of disagreement, a third party intervened to assess it. Each patient was followed up for at least 1 year.

### 2.5. Statistical Analysis

All data were counted by SPSS 22.0 and R. Univariate analysis was performed using the chi-square test, *t*-test, or Kruskal-Wallis rank-sum test. Logistic regression analysis was used to estimate the multivariate-adjusted odds ratio (OR) and 95% confidence intervals (95% CI). Multivariate models included age, gender, dyslipidemia, diabetes, smoking, drinking, lipid levels, and blood glucose levels. Heterogeneity *P* was calculated by adding the CRP tertile × subgroup variable interaction term to the model. Probability values of *P* < 0.05 were considered statistically different.

## 3. Results

### 3.1. Patient Characteristics

Patient demographic information and vascular risk factors were recorded within 48 hours of admission.  Table 1 shows the baseline demographic and clinical characteristics of LAA patients based on CRP levels. A total of 200 patients with LAA were collected in this study, of which 134 (67.0%) were males, with a mean age of 63.9 years, and 66 (33.0%) were females, with a mean age of 68.4 years. The median plasma CRP level on admission was 2.90 mg/L (the tertile range of all patients: 1.70-5.46 mg/L). At 3-month follow-up, the mean mRS for these patients was 1.64, while at 6- and 12-month follow-up, their mean mRS was 1.57 and 1.71, respectively (Table 1).

### 3.2. CRP Levels and Clinical Outcomes

We considered mRS ≥ 2 as the occurrence of an adverse outcome. At 3-month follow-up, the incidence of adverse outcomes was 45.0% (CRP level, 7.89 vs. 12.59); at 6 months, it was 42.5% (CRP level, 7.64 vs. 13.69); at 12 months, a total of 43.5% of patients had adverse outcomes (CRP level, 7.68 vs. 13.02).

**Figure 1:**
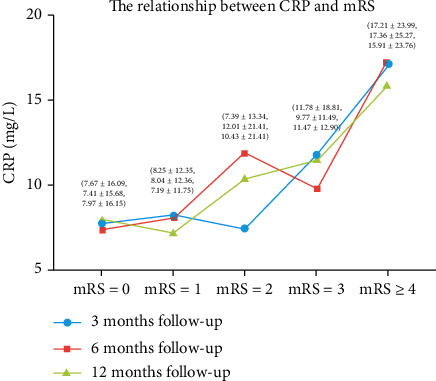
Plasma C-reactive protein levels and modified Rankin Scale. Note: at 3 months, 6 months, and 12 months of follow-up, CRP levels increased with increasing mRS. CRP: C-reactive protein; mRS: modified Rankin Scale.

**Figure 2:**
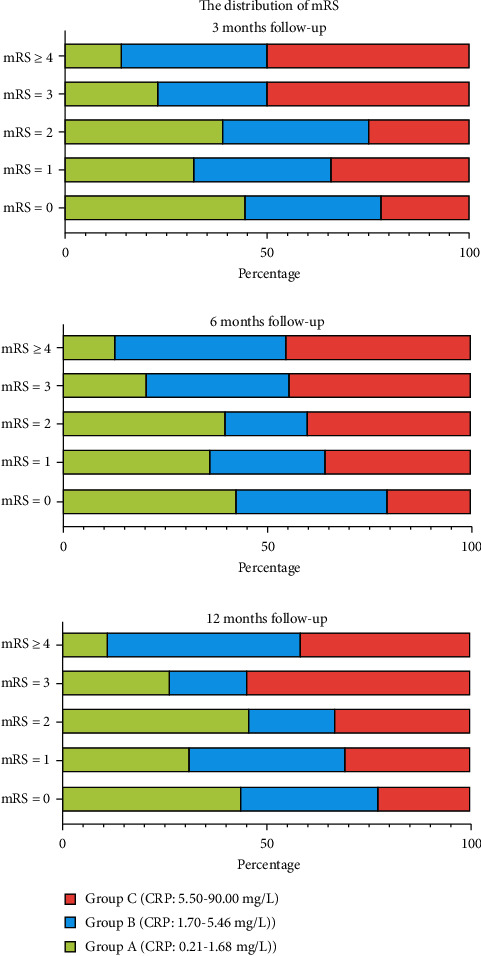
The percentage of three groups at different modified Rankin Scale scores.

In Figure 1, regardless of the time period of follow-up, when mRS was elevated, CRP levels overall also increased. However, at these three follow-up time points, when the mRS was the same, CRP levels did not show significant differences.  Figure 2 shows the percentage of each CRP level at different mRS levels. As mRS increased, the percentage of group C gradually increased.

**Figure 3:**
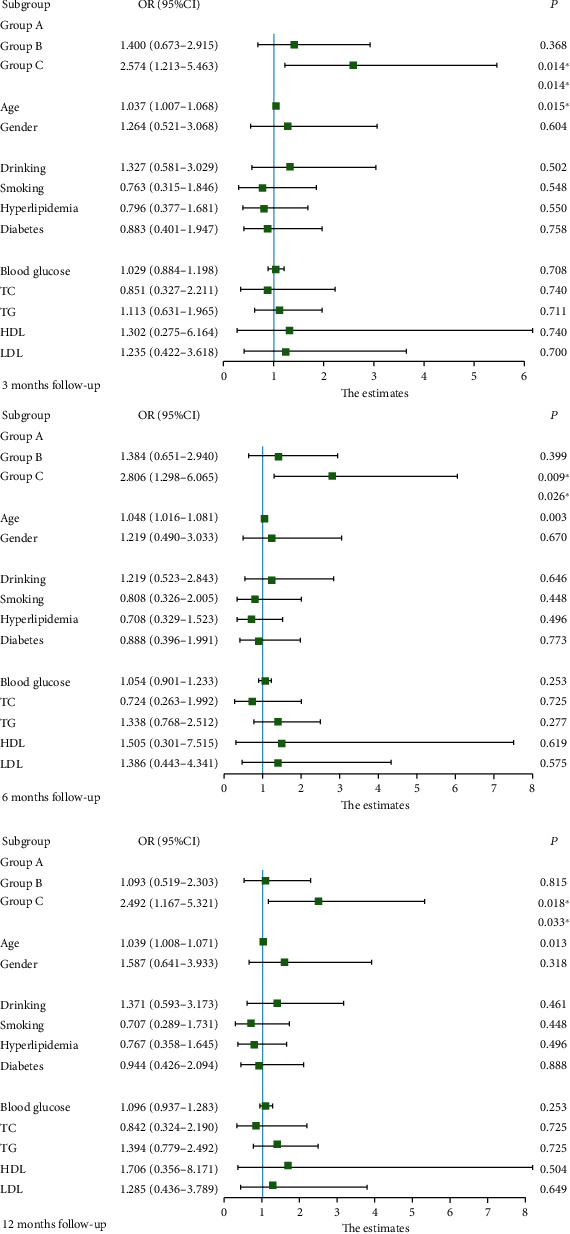
Multivariable-adjusted forest plot of each C-reactive protein tertile for poor functional outcome.

**Figure 4:**
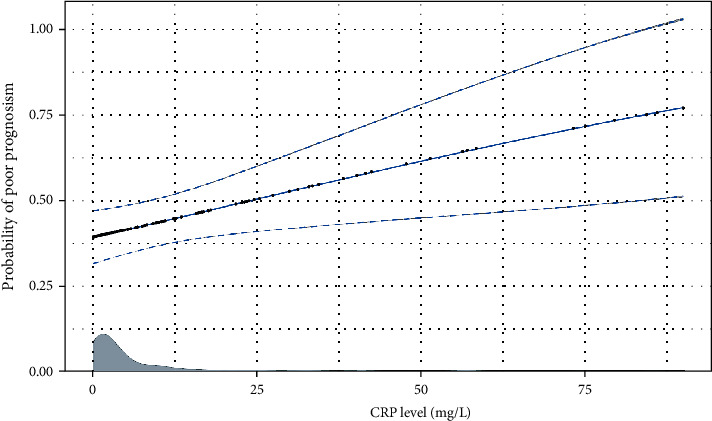
The linear regression of prognosis of C-reactive protein levels and acute ischemic stroke patients. Note: the abscissa represents C-reactive protein levels, and the ordinate shows the probability of poor prognosis. It can be seen that with the increase of C-reactive protein levels, the probability of poor prognosis is higher.

**Figure 5:**
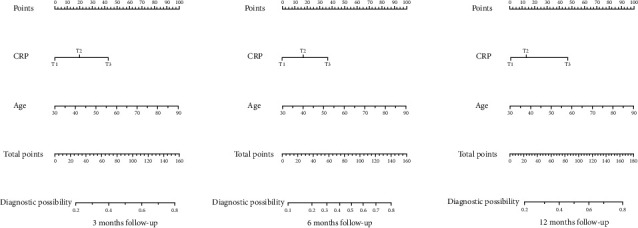
A nomogram model based on C-reactive protein and age. Note: the values on each variable axis have corresponding points, and the sum of these points on the survival axis has a probability of large artery atherosclerosis stroke corresponding to it.

After adjustment for gender, age, smoking history, drinking history, history of dyslipidemia, history of diabetes, lipid levels, and blood glucose levels, CRP levels were significantly associated with patient outcomes (Table 2). In a multifactorial logistic regression analysis, we found that the age of the patients was also a risk factor for adverse outcomes, and the older the patients, the more likely they were to have an adverse outcome. The patients' gender (*P* = 0.318), history of drinking (*P* = 0.461), history of smoking (*P* = 0.448), lipid level (*P* = 0.496), and blood glucose level (*P* = 0.888) were not significantly associated with the prognosis (Figure 3).  Figure 4 shows the linear region of diagnosis of CRP and AIS patients. Based on the two risk factors, CRP and age, we plotted a nomogram accordingly (Figure 5). Based on the nomogram, we can easily calculate the cumulative risk score of patients with LAA-type AIS and use this to calculate the corresponding probability of having an adverse outcome.

## 4. Discussion

In terms of disability and mortality, the major factors contributing to poor poststroke outcomes, such as age or baseline stroke severity, can not be modified. However, alterations in biomarkers may occur after stroke that may correlate with certain clinical outcomes, providing researchers and clinicians with a unique opportunity for interventions to improve stroke outcomes. Biomarkers serve as indicators of physiological or pathological biological processes that can be objectively measured and assessed. CRP is easily measured and commonly used and may be important in predicting functional prognosis after stroke [12]. Elevated CRP may reflect the degree of stroke, tissue damage, or systemic inflammatory response to concurrent infection [20]. The major finding of this study is that a higher level of CRP is an independent predictor of poor outcomes in patients with LAA-type AIS. Therefore, this study will give clinicians the insight that CRP is a widely available and easily accessible biomarker; early intervention with CRP in patients with LAA-type AIS may help these patients achieve a better clinical outcome.

Neuroinflammation is a unique pathophysiological feature of AIS patients [21, 22]. After an ischemic attack, the accumulation of inflammatory cells and mediators in the ischemic brain tissue triggers neuroinflammation. The inflammatory process is ignited by inflammatory cells such as leukocytes and activated microglia. A growing body of research suggests that CRP may be an inflammatory factor in response to ischemic stroke [23, 24]. Based on neuroinflammatory pathogenesis, patients with AIS are significantly associated with CRP [25]. Usually, in patients with severe stroke, CRP levels correlate with stroke severity and can be used as a marker of stroke prognosis [20, 26]. Studies have shown that the percentage of AIS patients with aortic sclerosis increases with increasing CRP levels; initial NIHSS scores, acute infection rates, and age increase with increasing CRP levels on admission; and the rate of neurological improvement increases with decreasing CRP levels [27]. CRP levels are elevated in the first 48 hours after onset and remain high for 3-6 months after stroke [18] [26].

In a meta-analysis, it was found that AIS patients with high CRP levels had an almost 2-fold increased risk of poor prognosis compared to patients with low CRP levels. This finding suggests that baseline CRP levels may predict the functional prognosis of patients after AIS [28]. Similarly, in our study, patients with the highest levels of CRP were 2.492 times more likely to have an adverse outcome than those with low levels of CRP at 12-month follow-up. In addition, a study found that at 90-day follow-up, an elevated each unit log-transformed CRP level was associated with a 2.5-fold higher risk of poor functional outcome [29]. In our 200 patients, at 90-day follow-up, high CRP was 2.574 times more likely to have an adverse outcome than patients with low CRP levels. Welsh et al. found that a one-unit increase in CRP levels was associated with an 18% increase in the risk of 1-year adverse functional outcome, and baseline CRP levels were a predictor of poor functional prognosis at 1 month postadmission [30].

A study found that artificial administration of CRP caused significantly greater infarcts than controls in a model of middle cerebral artery occlusion in adult rats [31]. In addition, in a rat model of acute myocardial infarction, artificial injection of CRP increased infarct size and exacerbated cardiac insufficiency, whereas the use of CRP inhibitors eliminated this effect [32]. These studies suggest that CRP has the inherent property of exacerbating ischemic injury. In patients with acute stroke, CRP was shown to be associated with poststroke functional outcome [32]. These studies suggest that intervening with CRP in patients early in stroke may improve ischemia-induced injury and shed new light on clinical treatment.

The paper still has several shortcomings. First, patients who were lost to follow-up were excluded, which may lead to selection bias. Second, our patient sample size remains inadequate, and single-center studies make the results insufficient for further generalization. Third, the follow-up period is one year; although it is longer than most similar studies, it is still insufficient. Stroke recurrence and patient death are very meaningful endpoint. However, this study is a small-sample prospective study with a follow-up time of 1 year. Due to the small sample size, the confidence interval in statistical analysis is extremely large, making the statistical results unreliable. However, we have been conducting a longer-term prospective study with a larger sample size, and we believe it can make up for this shortcoming.

## 5. Conclusion

High CRP levels in LAA-type AIS patients were associated with worse outcomes, both with short-term follow-up (3 months) and relative long-term follow-up (12 months). Early intervention on CRP levels in these patients may lead to more favorable outcomes and bring new thinking to the treatment of LAA-type AIS.

## Figures and Tables

**Table 1:** Characteristics of the cohort by C-reactive protein (CRP) levels.

	Group A (*n* = 66)	Group B (*n* = 67)	Group C (*n* = 67)
	CRP: <1.68	CRP: 1.70-5.46	CRP: >5.50
mRS 3 months	1.12 ± 1.34	1.63 ± 1.58	2.16 ± 1.68
mRS 6 months	1.06 ± 1.29	1.57 ± 1.63	2.06 ± 1.68
mRS 12 months	1.11 ± 1.31	1.73 ± 1.82	2.28 ± 1.97
NIHSS	3.26 ± 2.79	4.02 ± 3.72	5.81 ± 5.26
Age	64.06 ± 10.57	65.78 ± 11.59	66.34 ± 11.16
SBP	151.39 ± 20.51	163.54 ± 20.66	159.22 ± 24.69
DBP	80.41 ± 14.32	84.39 ± 13.03	82.16 ± 14.23
WBC	6.39 ± 1.53	6.70 ± 1.88	7.82 ± 2.59
Neutrophils	3.86 ± 1.38	4.14 ± 1.52	5.93 ± 6.72
Lymphocyte	1.89 ± 0.50	1.84 ± 0.56	1.76 ± 0.74
RBC	5.53 ± 0.52	4.61 ± 0.56	4.40 ± 0.61
PLT	208.35 ± 63.93	210.72 ± 60.57	241.32 ± 68.40
Albumin	38.33 ± 2.74	38.49 ± 4.08	36.91 ± 3.89
ALT	22.42 ± 12.33	22.18 ± 11.82	27.61 ± 45.50
AST	23.00 ± 9.29	24.16 ± 8.13	25.42 ± 12.99
Bilirubin	11.11 ± 4.02	11.54 ± 4.96	12.03 ± 6.85
Cr	69.15 ± 19.68	76.31 ± 38.45	69.61 ± 18.77
Bun	4.73 ± 1.92	5.80 ± 5.31	5.18 ± 1.69
TC	4.52 ± 1.00	4.60 ± 1.29	4.65 ± 1.35
TG	1.76 ± 0.81	1.83 ± 0.76	1.74 ± 1.06
HDL	1.06 ± 0.26	1.06 ± 0.24	1.06 ± 0.39
LDL	2.65 ± 0.80	2.72 ± 0.90	2.74 ± 1.01
Blood glucose	6.47 ± 1.60	6.60 ± 1.62	6.73 ± 1.61
Thyroxine	1.22 ± 0.24	1.23 ± 0.23	1.08 ± 0.29
Gender (female)	71.20%	67.20%	62.70%
DM	33.30%	37.30%	53.70%
Hyperlipidemia	54.50%	56.70%	70.10%
Smoking	57.60%	44.80%	40.30%
Drinking	47.00%	37.30%	31.10%

Abbreviation: NIHSS: the National Institutes of Health Stroke Scale; SBP: systolic pressure; DBP: diastolic pressure; WBC: white blood cell; RBC: red blood cell; PLT: platelet; AST: aspartate aminotransferase; ALT: alanine aminotransferase; Cr: creatinine; Bun: blood urea nitrogen; TC: total cholesterol; TG: triglyceride; HDL: high-density lipoprotein; LDL: low-density lipoprotein; DM: diabetes mellitus.

**Table 2:** Plasma C-reactive protein levels and functional outcomes during follow-up.

		Model 1			Model 2			Model 3		
	Events%	OR	95% CI	*P*	OR	95% CI	*P*	OR	95% CI	*P*
Outcome 1 (3-month follow-up)										
Group A	33.33%	Ref	Ref	Ref	Ref	Ref	Ref	Ref	Ref	Ref
Group B	44.78%	1.559	0.762-3.192	0.224	1.548	0.752-3.187	0.236	1.400	0.673-2.915	0.368
Group C	56.72%	2.545	1.241-5.221	0.011^∗^	2.611	1.241-5.496	0.011^∗^	2.574	1.213-5.463	0.014^∗^
*P*				0.038^∗^			0.040^∗^			0.044^∗^
Outcome 2 (6-month follow-up)										
Group A	30.30%	Ref	Ref	Ref	Ref	Ref	Ref	Ref	Ref	Ref
Group B	41.79%	1.572	0.754-3.276	0.228	1.553	0.741-3.253	0.243	1.384	0.651-2.940	0.399
Group C	55.22%	2.756	1.324-5.734	0.007^∗^	2.751	1.290-5.865	0.009^∗^	2.806	1.298-6.065	0.009^∗^
*P*				<0.001^∗^			0.031^∗^			0.026^∗^
Outcome 3 (12-month follow-up)										
Group A	33.33%%	Ref	Ref	Ref	Ref	Ref	Ref	Ref	Ref	Ref
Group B	40.30%%	1.299	0.632-2.669	0.476	1.267	0.611-2.626	0.524	1.093	0.519-2.303	0.815
Group C	56.72%%	2.57	1.253-5.269	0.010^∗^	2.446	1.165-5.135	0.018^∗^	2.492	1.167-5.321	0.018^∗^
*P*				0.027^∗^			0.047^∗^			0.033^∗^

Note: in the logistic regression analysis, Model 1 adjusted for gender and age. Model 2 incorporates gender, age, smoking history, drinking history, history of dyslipidemia, and history of diabetes as confounding factors, and Model 3 incorporates lipid levels and blood glucose levels on the basis of Model 2. We can see that after adjusting for various confounding factors, CRP still significantly affects the prognosis of patients.

## Data Availability

The raw data used to support the findings of this study are made available from the corresponding author upon reasonable request.
